# A Polymeric Prodrug of 5-Fluorouracil-1-Acetic Acid Using a Multi-Hydroxyl Polyethylene Glycol Derivative as the Drug Carrier

**DOI:** 10.1371/journal.pone.0112888

**Published:** 2014-11-12

**Authors:** Man Li, Zhen Liang, Xun Sun, Tao Gong, Zhirong Zhang

**Affiliations:** Key Laboratory of Drug Targeting and Drug Delivery Systems, Ministry of Education, West China School of Pharmacy, Sichuan University, Chengdu, Sichuan, PR China; Laval University Cancer Research Centre, Canada

## Abstract

**Purpose:**

Macromolecular prodrugs obtained by covalently conjugating small molecular drugs with polymeric carriers were proven to accomplish controlled and sustained release of the therapeutic agents *in vitro* and *in vivo*. Polyethylene glycol (PEG) has been extensively used due to its low toxicity, low immunogenicity and high biocompatibility. However, for linear PEG macromolecules, the number of available hydroxyl groups for drug coupling does not change with the length of polymeric chain, which limits the application of PEG for drug conjugation purposes. To increase the drug loading and prolong the retention time of 5-fluorouracil (5-Fu), a macromolecular prodrug of 5-Fu, 5-fluorouracil-1 acid-PAE derivative (5-FA-PAE) was synthesized and tested for the antitumor activity *in vivo*.

**Methods:**

PEG with a molecular weight of 38 kDa was selected to synthesize the *multi-hydroxyl polyethylene glycol* derivative (PAE) through an addition reaction. 5-fluorouracil-1 acetic acid (5-FA), a 5-Fu derivative was coupled with PEG derivatives via ester bond to form a macromolecular prodrug, 5-FA-PAE. The *in vitro* drug release, pharmacokinetics, *in vivo* distribution and antitumor effect of the prodrug were investigated, respectively.

**Results:**

The PEG-based prodrug obtained in this study possessed an exceedingly high 5-FA loading efficiency of 10.58%, much higher than the maximum drug loading efficiency of unmodified PEG with the same molecular weight, which was 0.98% theoretically. Furthermore, 5-FA-PAE exhibited suitable sustained release in tumors.

**Conclusion:**

This study provides a new approach for the development of the delivery to tumors of anticancer agents with PEG derivatives.

## Introduction

Cancer is one of the most life-threatening diseases worldwide, which seriously endangers human health and survival [1], [2]. Surgery, radiotherapy, chemical medication, biological immunization therapies are the major treatment strategies, among which chemotherapy plays an important role in the treatment of cancer [3]–[9]. Regarding chemotherapies, 5-fluorouracil (5-Fu) is one of the most widely used antimetabolites in clinic [10], which shows significant inhibitory effect against a broad spectrum of solid tumors [11]–[13]. Traditional chemotherapies such as 5-Fu are cytotoxic agents that inhibit rapidly proliferating cancer cells. Due to its low specificity, side effects such as myelosuppression, mucositis, dermatitis and diarrhea are commonly observed during the clinical application of 5-Fu [14]–[16]. Additionally, 5-Fu has a very short half life of about 20 minutes and is rapidly eliminated after administration. The irregular oral absorption and the low bioavailability often results in poor clinical therapeutic outcome [17]–[19].

To address the aforementioned problems, researchers have tried various methods to improve the efficacy and to reduce the toxicity of 5-Fu, including modification of the chemical structure, formulation strategies and novel delivery systems. Several small molecular prodrugs of 5-Fu were developed, such as 5-fluoro-2′-deoxyuridine, 1-(2-tetrahydrofuryl)-5-fluorouracil and 3, 5-dioctanoyl 5-fluoro-2-deoxyuridine [20]–[22]. Various delivery systems have been developed for the targeted delivery of 5-Fu [23]. Menei *et al* developed biodegradable microspheres to obtain sustained delivery of 5-Fu for the treatment of glioblastoma [24]. Liposomes have been used as a sustained delivery system for 5-Fu [25]. In recent years, macromolecular carrier/delivery systems have been studied extensively. Macromolecular prodrugs obtained by combining small molecular drugs with polymeric carriers could slowly release the therapeutic agents *in vivo* with an improved half-life [26]–[31]. Moreover, the enhanced permeability and retention (EPR) effect may contribute to the accumulation of macromolecular prodrugs within the solid tumor, which would lead to a tumor-targeted drug delivery and reduced toxicity to normal tissues [32]–[34]. Moreover, the EPR effect has been regarded as the “golden rule” in the design of antitumor drugs. Based on the EPR effect, numerous tumor-targeted drug delivery systems were developed using macromolecules such as albumin (65 kDa), transferrin (90 kDa), IgG (immunoglobulin, 150 kDa), α2-macroglobulin (240 kDa) and ovomucoid of chicken eggwhite (29 kDa, highly glycosylated protein), and some have entered clinical trials [35].

In addition to the aforementioned macromolecular materials, polyethylene glycol (PEG) has become a material of great interests due to its low toxicity, low immunogenicity and high biocompatibility [36]–[38]. The molecular weight of PEG used in forming macromolecular prodrugs would impact the *in vivo* behaviors of the conjugates because the retention time of the prodrugs increased with the molecular weight of the carriers [30]. Prolonged retention of the prodrug is critical to the tumor accumulation of the therapeutic agents loaded. However, for linear PEG macromolecules, the number of available hydroxyl groups for drug coupling does not change with the length of the polymeric chain, which limits the application of PEG for drug conjugation purposes. Therefore,the development of new PEG derivatives to improve its drug loading efficiency has become a hot topic in material science and is of great significance to the tumor-targeted delivery of small molecular agents and 4-arm PEG derivatives were thus developed [39], and the 4-arm PEG based prodrugs have entered clinical trials with promising results [40]–[44]. For small molecular drugs such as 5-Fu, treatment requires a high therapeutic concentration, while the macromolecular based prodrugs have a relatively low drug loading efficiency. Thus, the modification of linear PEG creates derivatives with high drug loading efficiency which will have great significance for anticancer drug development [45].

In this study, a macromolecular prodrug, 5-fluorouracil-1 acid- PAE derivative (5-FA-PAE), was designed and synthesized to increase the drug loading efficiency, achieve delivery to the tumor and prolong the retention time. PEG with a molecular weight of 38 kDa was selected as the starting material to obtain the multi-hydroxyl PEG derivative, which was then coupled with 5-fluorouracil-1 acetic acid (5-FA), to afford the prodrug. The *in vitro* drug release, pharmacokinetics, *in vivo* distribution and antitumor effect of the prodrug were investigated, respectively.

## Materials and Methods

### Materials

Polyethylene glycol (PEG, average molecular weight ∼38 kDa), allyl glycidyl ether (AGE), mercaptoethanol, 1-(3-dimethylaminopropyl)-3-ethylcarbodiimide hydrochloride (EDC·HCl) and N-hydroxysuccinimide (NHS) were purchased from Sigma-Aldrich (USA). Sodium hydride (NaH) was supplied by Damao Chemical Reagent Factory (Tianjin, China). 5-Fluorouracil (USP29) was purchased from Nantong Jinghua Pharmaceuticals Co., Ltd (Jiangsu, China). All other chemicals used were of reagent grade.

### Synthesis of multi-hydroxyl polyethylene glycol derivative (polyethylene glycol-allyl glycidyl ether-mercaptoethanol, PAE)

Polyethylene glycol-allyl glycidyl ether (PA) was synthesized as described before [46], [47] with some modifications. Briefly, 10.0 g of PEG was melted in an oil bath at 120°C with stirring under vacuum for about 3 h to remove the adsorbed moisture before adding 120 mg of NaH. The mixture was stirred for 4 h at 120°C, and 2.0 ml of AGE was added. The product was recrystallized with isopropanol to remove the micromolecular materials.

### Synthesis of 5-FA-PAE prodrug

5-FA was synthesized as previously described [48], [49] with some modification. Briefly, 6.5 g of 5-Fu was dissolved in 25 ml of aqueous solution of potassium hydroxide (4 M), then 15 ml of aqueous solution of chloroacetic acid (5 M) was added dropwise with stirring. The pH value of the reaction mixture was monitored and kept at 10 by adding an aqueous solution of potassium hydroxide (10 M) during the addition of chloroacetic acid and throughout the whole course of the reaction. The mixture was heated to 50°C in an oil bath with stirring for 8 h, and then acidified by HCl to obtain 5-FA.

A solution of 5-FA (0.496 g) in 1 ml of dimethylformamide was added dropwise to a solution of 0.5 g of PAE in 20 ml of dimethylformamide, then 0.196 g (1.7 mmol) of NHS and 0.4 g (2.09 mmol) of EDC·HCl were added sequentially. After a further 16 h of incubation at room temperature away from light, the mixture was precipitated with 150 ml of isopropanol. The obtained residue was recrystallized by isopropanol several times until the reagents and uncoupled 5-FA were totally removed (monitored by TLC and HPLC), then dried in vacuum at 40°C overnight.

### HPLC analysis

HPLC assay was established for the determination of 5-FA in PBS, plasma or tissues homogenates, which was performed using Shimadzu instruments (Chiyoda-Ku, Japan) consisting of a CTO-10A column thermostat, two LC-10AT pumps and a SPD-10A UV detector. A Scienhome ODS column (5 µm, 150×4.6 mm, Tianjin, China) was used to separate samples. Phosphate buffer (0.05 M, pH 2.5) was used as the mobile phase at a flow rate of 1 ml/min. The temperature of the column was kept at 35°C and the effluent was detected at 270 nm. Studies showed that the precision, accuracy, and recovery of this HPLC method all met the measurement requirements.

### Safety evaluation

All animal experiments were approved by the Institutional Animal Care and Ethic Committee of Sichuan University (Approved No. SYXK2013-185). All animals were fed on a light and dark cycle and allowed free access to standard chow and water. Temperature and relative humidity were kept at 25°C and 50%, respectively. After experiment, mice were sacrificed by neck dislocation, and all efforts were made to minimize suffering. Myelosuppression is one of the major side effects of 5-Fu [14]. To assess the suppression level, 60 male Kunming mice (20–25 g, purchased from Laboratory Animal Center of Sichuan University) were randomly divided into 5 groups (n = 12) and were intravenously administered with 5-Fu (27.66 mg/kg), 5-FA (40 mg/kg), PAE (338 mg/kg) or 5-FA-PAE (378 mg/kg) (equivalent to 0.213 mmol/kg 5-FA). The control group was given physiological saline (0.009 g/ml). Zero point one mL blood samples were collected at prearranged time intervals (one day before injection and 1, 4, 7, and 10 days post injection). The white blood cells (WBC) and the blood platelets number were counted by MEK-6318K Automated Hematology Analyzer (Nihonkohden, Shinjuku-ku, Japan) as an index of myelosuppression.

### *In vitro* drug release

The *in vitro* drug release of 5-FA-PAE was investigated in physiological saline (0.009 g/ml), PBS with various pH values, 50% mouse plasma (diluted with PBS, pH 7.4, v/v) and 50% mouse tumor homogenate which was obtained from the H22 tumor loaded mice (homogenized and diluted with physiological saline). An aqueous solution of 5-FA-PAE (100 µl) was added to 4 ml of preheated release medium (physiological saline or PBS with pH = 3.04, 4.51, 6.02, 7.41, 8.99). The mixture was maintained in a water bath at 37°C under continuously stirring, and 100 µl of each sample was collected at fixed time intervals (i.e. 0.25, 1, 3, 6, 10, 24, 48, 72, 96 h). The samples from physiological saline and PBS was acidified by 100 µl hydrochloric acid (1 M), diluted with 300 µl mobile phase and analyzed by HPLC. The samples from mouse plasma and tumor homogenate were obtained in duplicate at each time point (100 µl each). For hydrolysis, samples were mixed with 50 µl of aqueous solution of 5-bromouracil (96 µg/ml, 50 µl) as the internal standard, and then supplemented with 100 µl sodium hydroxide (1 M) and acidified by 100 µl hydrochloric acid (1 M), and extracted by 3.3 ml of ethyl ester for 15 min. After centrifugation at 10,000 rpm for 5 min, 2.7 ml of the ethyl ester portion was collected, concentrated in a nitrogen gas flow, redissolved in 100 µl of the mobile phase and centrifuged at 10,000 rpm for 10 min before HPLC analysis. The other group was not subjected to hydrolysis by substituting sodium hydroxide solution with saline and acidifying with 50 µl hydrochloric acid. The differences of 5-FA in the two groups at the same time point was the unreleased 5-FA in each sample. The decrement method was used to calculate the release rate. All experiments were conducted in triplicate.

### Pharmacokinetics study

Male Wistar rats were purchased from The laboratory Animal Center of Sichuan University. 12 Wistar rats (body weight: 200 g±20 g) were divided into two groups randomly (n = 6). The control group and the test group were administered intravenously with 20 mg/kg of 5-FA and 189 mg/kg 5-FA-PAE (equivalent to 20 mg/kg of 5-FA) dissolved in physiological saline, respectively. The blood samples were collected into heparinized centrifuge tubes at predetermined intervals (see Table S2 in file SI) by retro-orbital puncture, and the plasma was separated by centrifugation. Each plasma sample of the test groups was divided into two portions. They were treated as hydrolyzed and unhydrolyzed as described in the “*In vitro* drug release” section. The two portions of the samples were analyzed by HPLC to determine the plasma concentrations of released 5-FA and total 5-FA of the conjugate whereas the plasma samples of the control group were treated as unhydrolyzed samples.

### *In vivo* distribution

Murine H22 hepatocarcinoma cells (purchased from Type Culture Collection of Chinese Academy of Sciences) were maintained in RPMI 1640 medium supplemented with 2 mM L-glutamine and 10% fetal bovine serum (FBS) at 37°C with 5% CO_2_, and were passaged every 2 or 3 days. The tumor-bearing animal model was established by subcutaneous injection of H22 cells (1×10^7^ cells/ml, in 0.2 ml saline) into the right axillary region of Kunming mice. The sizes of tumors were monitored 7 days after inoculation and the tumor volumes were calculated as described in the “*Antitumor activity in tumor-bearing mice*” section. The mice with tumor volumes between 0.35 cm^3^ and 0.65 cm^3^ were randomized into two groups (n = 30). The control group and the test groups were administered intravenously with 20 mg/kg of 5-FA or 189 mg/kg 5-FA-PAE (equivalent to 20 mg/kg of 5-FA) dissolved in physiological saline (0.009 g/ml), respectively. The mice were exsanguinated and sacrificed by neck dislocation at predetermined time points. Tissues including heart, liver, spleen, lung, kidney, brain and tumor were collected, washed with physiological saline, weighed and homogenized with two fold concentrated physiological saline. The samples of the test group were treated as hydrolyzed samples as described in the section “*In vitro* drug release”, whereas those of the control group were treated as unhydrolyzed samples. All data are presented as the concentration of 5-FA.

### Antitumor activity in tumor-bearing mice

The tumor-bearing mice model was established as previously described in the “*In vivo distribution*” section. 72 h after inoculation, mice with no signs of tumor growth were exclude from this experiment. 48 tumor-bearing mice were randomly divided into 4 groups (n = 12). The control group was administered intravenously with 20 ml/kg of physiological saline. The other groups were administered intravenously with 30 mg/kg (0.160 mmol/kg) of 5-FA or 284 mg/kg 5-FA-PAE (equivalent to 0.160 mmol/kg 5-FA) dissolved in physiological saline. 5-Fu (20.47 mg/kg, 0.160 mmol/kg) was administered as a control. All animals were administered once on day 3, 5, 7, 9, 11, 13, 15 after the inoculation of H22 cells and sacrificed on day 20. Tumors and organs (heart, liver, spleen, lung, kidney, brain and thymus) were removed and weighed. The tumor volume and tumor control rate were evaluated. The tumor volume, organ/body weight index and tumor control rate were calculated as follows:
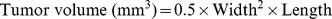


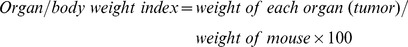


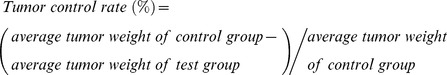



### Data analysis

The data of pharmacokinetics and *in vivo* distribution study were processed using the Drug and Statistics Software 2.0 (DAS 2.0, Shanghai, China). The statistical analysis of the samples was performed by using one-way ANOVA and Student's *t*-test. *p*-values <0.05 were considered as statistically different.

## Results and Discussion

### Synthesis and characterization of 5-FA-PAE prodrug

**Figure 1:**
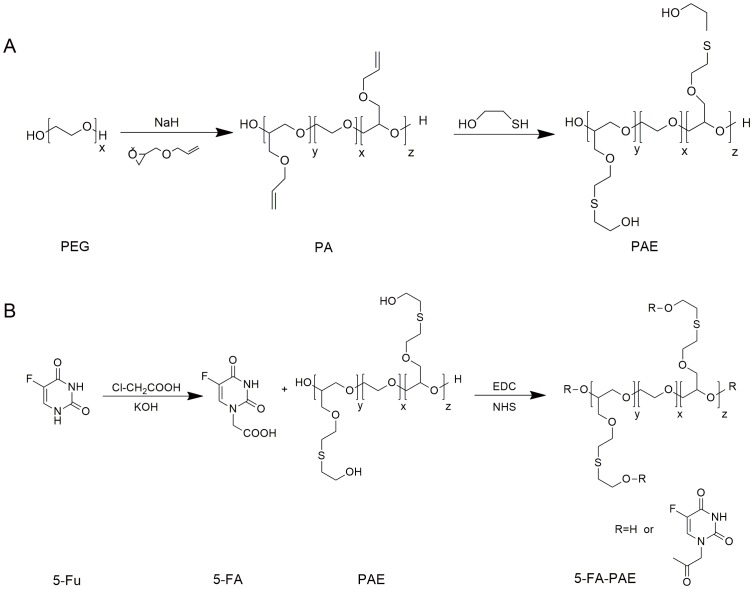
Synthesis routes of PAE (A) and 5-FA-PAE conjugates (B). (A) PA was synthesized by adding NaH to PEG and the mixture was stirred for 4 h at 120°C. Then PAE was obtained by adding AGE to the mixture. (B) 5-FA was added dropwise to PAE in dimethylformamide, then NHS and EDC·HCl were added. After incubation, the mixture was precipitated with isopropanol. The obtained residue was recrystallized by isopropanol several times and dried in vacuum at 40°C overnight to produce 5-FA-PAE.

As a polyether macromolecule, PEG is widely used for its suitable solubility and bioavailability in developing drug delivery systems [50]–[53]. However, as a drug carrier, the loading efficiency of prodrugs based on PEG is significantly constrained due to the limited positions for drug conjugation, *i.e.*, two hydroxy groups in the linear PEG molecule [45]. Thus, the modification of PEG to create derivatives with higher drug loading efficiency is greatly needed. PEG with a molecular weight of 38 kDa was selected as the starting material to synthesize the derivative (Fig. 1A). Allyl glycidyl ether was coupled to both ends of PEG under the catalysis of sodium hydride to form an intermediate, namely PA, with multi-double bonds on the side chains. PA was further reacted with small molecules through the addition reaction of the double bonds and the thiol group to afford various PEG derivatives with multi-hydroxyl groups. ^1^H-NMR showed that the double bonds disappeared completely in PAE (Figure S1 in file SI). The GPC analysis demonstrated that PA and PAE had similar molecular weight distribution as the starting material PEG (Table 1). As a common drug carrier, the molecular weight of PEG greatly influenced the *in vivo* behaviors of prodrugs [54]. As the molecular weight increases, the *in vivo* clearance rate decreases. Thus, PEG with a higher Mw is likely to prolong the retention time of prodrugs and increase the drug accumulation in a tumor. It is suggested that the Mw of PEG should be no less than 30 kD to prevent the prodrug from quick elimination from kidney [55]. Accordingly, PEG of 38 kD was used as the starting material.

**Table 1:** The molecular weight of the polymeric carrier (PEG, PA, PAE) and the prodrug (5-FA-PAE).

Compound	MP (Da)	Mw (Da)	Mn (Da)	PDI
PEG	38914	38045	27596	1.38
PA	33984	36039	21322	1.69
PAE	34282	36628	20842	1.76
5-FA-PAE	35413	44629	19998	2.23

PDI: polydispersity.

However, 5-Fu could not be directly coupled with the carriers due to the lack of available hydroxyl groups in the structure. The derivative of 5-Fu, 5-fluorouracil-1-acetic acid (5-FA), was synthesized first. The macromolecular prodrug multi-hydroxyl polyethylene glycol-5-fluorouracil-1-acetic acid (5-FA-PAE) was obtained by covalently conjugating 5-FA with the PEG derivative under the catalysis of carbodiimide condensing agents (Figure 1B). The successful synthesis of the 5-fluorouracil derivative and the prodrug was confirmed by ^1^H-NMR (Figure S1 in file SI). A higher molecular weight (Mw) and polydispersity (PDI) of 5-FA-PAE were observed compared with those of PAE (Table 1). HPLC analysis indicated that after the double bond-thiol addition reaction, multiple hydroxyl groups were introduced on the PEG backbone thus making it capable of loading more drugs. The drug loading efficiency of 5-FA-PAE was determined as 10.58%, much higher than the maximum drug loading efficiency of PEG with the same molecular weight, which was calculated as 0.98% theoretically. The drug loading efficiency of 5-FA-PAE was improved by 10.8-fold compared to that of 5-FA. The significant enhancement in drug loading efficiency would greatly increase the drug concentration within a tumor via the EPR effect.

### Safety evaluation

To investigate the myelosuppression levels after 5-FA or 5-FA-PAE treatment, hematological parameters (i.e. the number of white blood cells and blood platelets) were measured at different time points after drug administration. Changes in these parameters presumably reflect the occurrence of myelosuppression and abnormality in the immune system. As shown in Table 2, the WBC count decreased after intravenous injection of 5-Fu, 5-FA and 5-FA-PAE. Only the group of 5-Fu exhibited significant reduction of WBC (5.39±2.17×10^9^/L one day after injection, *p*<0.05). Then the WBC level increased gradually. Notably, the increase of WBC in the 5-Fu group was slower, leading to a lower WBC level at day 10 (8.59±2.39×10^9^/L) compared with the level before administration (9.55±1.28×10^9^/L), while the WBC level of other groups recovered within 4 days. Another major indicator of myelosuppression is the change in blood platelets number. Table S1 in file SI shows that though the platelets number of 5-Fu decreased 1 day after injection, the blood platelets of all groups didn't exhibit any significant changes, indicating that both 5-FA and 5-FA-PAE hardly affect the platelets level at such doses. Taken together, these results indicated that the prodrug of 5-Fu, 5-FA-PAE, showed a lower toxicity than 5-Fu.

**Table 2:** The number of white blood cells in mice administered with saline, 5-Fu, 5-FA, PAE or 5-FA-PAE (×10^9^/L).

	1 day before injection	1 day after injection	4 days after injection	7 days after injection	10 days after injection
saline	9.77±1.99	8.86±2.79	11.19±6.78	12.24±4.37	12.06±7.58
5-FA-PAE	9.35±1.39	8.73±1.90	11.73±5.44	12.00±2.02	12.52±3.78
5-FA	9.13±2.23	8.99±1.86	10.32±5.71	10.24±4.50	11.55±3.62
PAE	9.54±1.64	8.46±1.85	9.85±3.09	10.74±5.13	11.93±3.40
5-Fu	9.55±1.28	5.39±2.17*	6.84±5.76	7.34±2.82	8.59±2.39

Each value represents the mean ± SD (n = 12).

*p<0.05 vs. 5-Fu,1 day before injection.

### *In vitro* drug release

**Figure 2:**
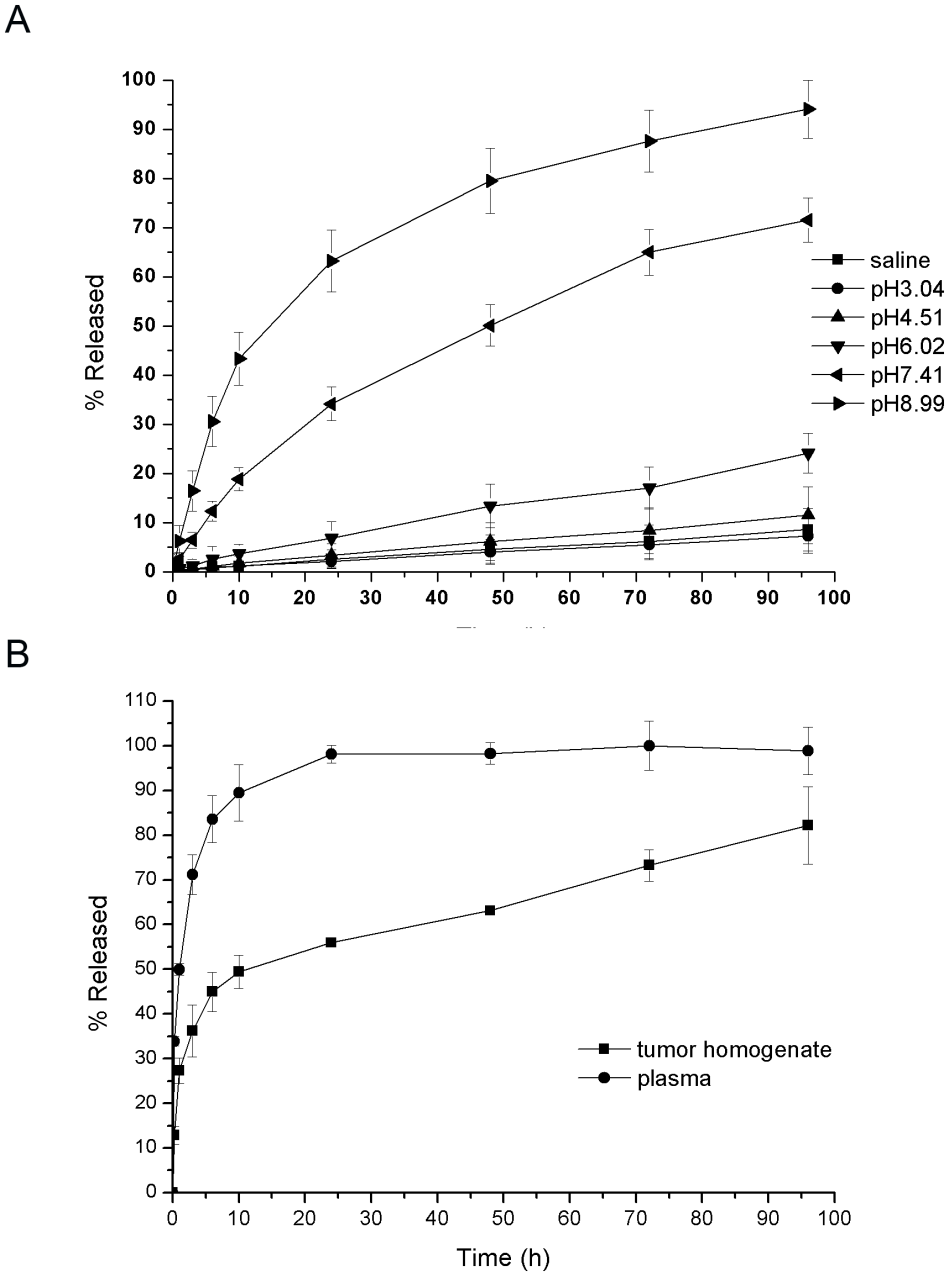
*In vitro* drug release of 5-FA-PAE. (A) Drug release profiles of 5-FA-PAE in PBS and saline. 100 µl 5-PA-PAE was added to preheated release media (PBS of different pH values or saline) and incubated at 37°C with stirring. Samples were collected at fixed time intervals, acidified by hydrochloric acid (1 M) and analyzed by HPLC. (B) Drug release profiles of 5-FA-PAE in murine tumor homogenate and plasma. The samples from mouse plasma and tumor homogenate were obtained in duplicate at each time point (100 µl each). For hydrolysis, 100 µl sodium hydroxide (1 M) were added to samples followed by 100 µl hydrochloric acid (1 M). The other group was not subjected to hydrolysis by substituting sodium hydroxide solution with saline and acidifying with 50 µl hydrochloric acid. The differences of 5-FA in the two groups at the same time point was the unreleased 5-FA in each sample. Each value represents the mean ± SD (n = 3).

The *in vitro* drug release behavior of 5-FA-PAE was investigated using phosphate buffered saline (PBS) of various pH values, physiological saline, mouse plasma and tumor homogenate as the release media. The release rate of 5-FA-PAE was pH-dependent. As the pH increased, the release rate increased significantly, reaching 94.1%±5.88% at 96 h when the pH was 8.99 (Figure 2A). This is mostly likely due to the hydrolysis of ester bonds under basic conditions. However, if the conjugation with the PEG derivative increased the retention time in plasma, this would possibly enhance the drug accumulation in a tumor (Figure 2B). The release rate of prodrug 5-FA-PAE in plasma was 89.46%±6.36% at 10 h, and reached 98.15%±1.96% at 24 h, while the rate was 55.9%±0.61% in tumor homogenate at 24 h, suggesting that the ester bond can be easily degraded by easterases in plasma.

### Pharmacokinetics study

**Figure 3:**
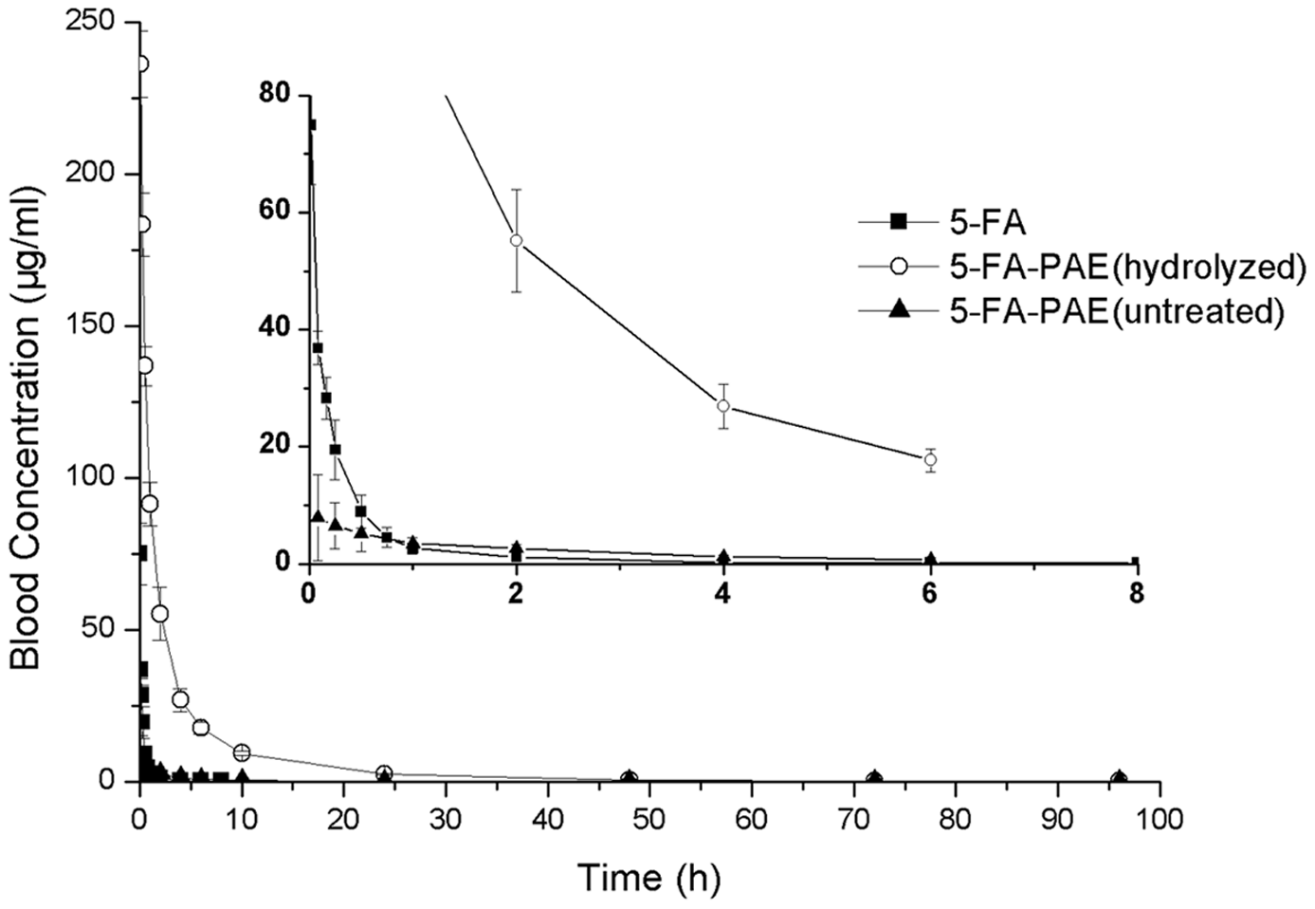
Pharmacokinetics of 5-FA and 5-FA-PAE after *i.v.* injection. The control group and the test groups were administered intravenously with 20 mg/kg of 5-FA or 189 mg/kg of 5-FA-PAE (equivalent to 20 mg/kg of 5-FA) dissolved in physiological saline, respectively. Each plasma sample of the 5-FA-PAE group was divided into two portions (treated as hydrolyzed and unhydrolyzed), which were analyzed by HPLC to determine the plasma concentrations of released 5-FA and total 5-FA of the conjugate whereas the plasma samples of the control group were treated as unhydrolyzed samples. Each value represents the mean ± standard deviation (n = 6).

A major drawback of 5-Fu is the relatively short half-life, which results in poor patient compliance and side effects. 5-FA, the derivative of 5-Fu, shows the same metabolism and clearance rate as 5-Fu. Moreover, after conjugation with PEG, the retention time in plasma was greatly prolonged, which might enhance tumor accumulation. After administration intravenously, 5-FA was rapidly eliminated from the blood circulation, which led to a complete removal at 6∼8 h after administration, whereas the elimination rate of 5-FA-PAE was much lower than that of 5-FA, and the blood retention time of this macromolecular prodrug reached more than 96 h (Figure 3). The detailed plasma concentration of 5-FA and 5-FA-PAE at different time points are shown in Table S2 in file SI. Some pharmacokinetic parameters, such as the area under the curve (AUC), the mean retention time (MRT) and the elimination half-life (t_1/2_) of 5-FA-PAE were much higher than those of 5-FA, *i.e.*, 25.6 times for AUC (546.6±36.7 µg/ml ·h vs 21.37±4.36 µg/ml ·h), 11.7 times for MRT (7.962±0.400 h vs 0.679±0.142 h) and 14.4 times for t_1/2_ (22.10±5.92 h vs 1.538±0.419 h), indicating a much longer blood circulation times and a remarkably enhanced bioavailability of the macromolecular prodrug (Table 3). Meanwhile, the amount of 5-FA released from 5-FA-PAE in rat plasma was determined. Although the total amount of 5-FA-PAE in rat plasma was significantly higher than that of 5-FA, the concentration of released 5-FA from 5-FA-PAE was not as much as 5-FA. It was lower than the 5-FA group within 30 min after administration and then increased slightly afterwards (Table S2 in file SI). This may have an impact on the antitumor efficacy of 5-FA-PAE, but could avoid certain possible side effects.

**Table 3:** Pharmacokinetic parameters of 5-FA and 5-FA-PAE after i.v. injection in rats.

Parameters	Unit	5-FA	5-FA-PAE
AUC _0-t_	µg/ml·h	21.37±4.36	546.6±36.7
AUC _0-∞_	µg/ml·h	21.63±4.47	551.5±35.0
MRT _0-t_	h	0.679±0.142	7.962±0.400
MRT _0-∞_	h	0.795±0.240	9.072±0.513
VRT _0-t_	h^2^	1.519±0.326	190.2±19.7
VRT _0-∞_	h^2^	2.703±1.558	335.8±79.4
t_1/2_	h	1.538±0.419	22.10±5.92
T_max_	h	0.0167±0	0.097±0.034
V	ml/g	2.107±0.620	1.172±0.358
Cl	ml/g/h	0.967±0.259	0.036±0.002
C_max_	µg/ml	74.92±10.16	232.4±13.5

AUC, area under the plasma concentration−time curve; MRT, mean residence time; VRT, variance of mean residence time; t_1/2_: elimination half life; T_max_, time of maximum concentration; V, apparent volume of distribution; CL, clearance; Cmax, the maximum of 5-FA concentration in plasma.

Each value represents the mean ± SD (n = 5).

Due to the obvious discrepancy of retention time between 5-FA and 5-FA-PAE, two sets of different time points were adopted to fully describe the *in vivo* fate of 5-FA and 5-FA-PAE. The first time point for 5-FA and 5-FA-PAE was 1 min and 5 min after administration, respectively.

In the *in vitro* release study, about 98% of 5-FA was released from 5-FA-PAE in plasma at 24 h, in other words, 2% 5-FA-PAE remained intact, while in the pharmacokinetics study, the concentration of unreleased 5-FA-PAE was 228.276±5.441 µg/ml 5 min after administration (the difference between total concentration of 5-FA-PAE and free 5-FA released from 5-FA-PAE), and decreased to 2.439±0.258 at 24 h (about 1.1% of the concentration at 5 min), which is consistent with the previous *in vitro* release study. Comparing the concentration of the free 5-FA of unhydrolyzed and hydrolyzed 5-FA-PAE group, it can be concluded that the unreleased 5-FA-PAE was intact in the blood circulation, which could accumulate in tumor tissue in the form of prodrug and then slowly release 5-FA at the tumor site to achieve antitumor effect. 5-FA was shown to be rapidly eliminated from blood, while after conjugation with PAE, the retention time was significantly prolonged, which may be attributed to a protective role of PEG. Thus, the prolonged retention time of 5-FA not only extended the duration time and enhanced the bioavailability, but also improved the delivery of macromolecular drugs to a tumor.

### *In vivo* distribution

**Figure 4:**
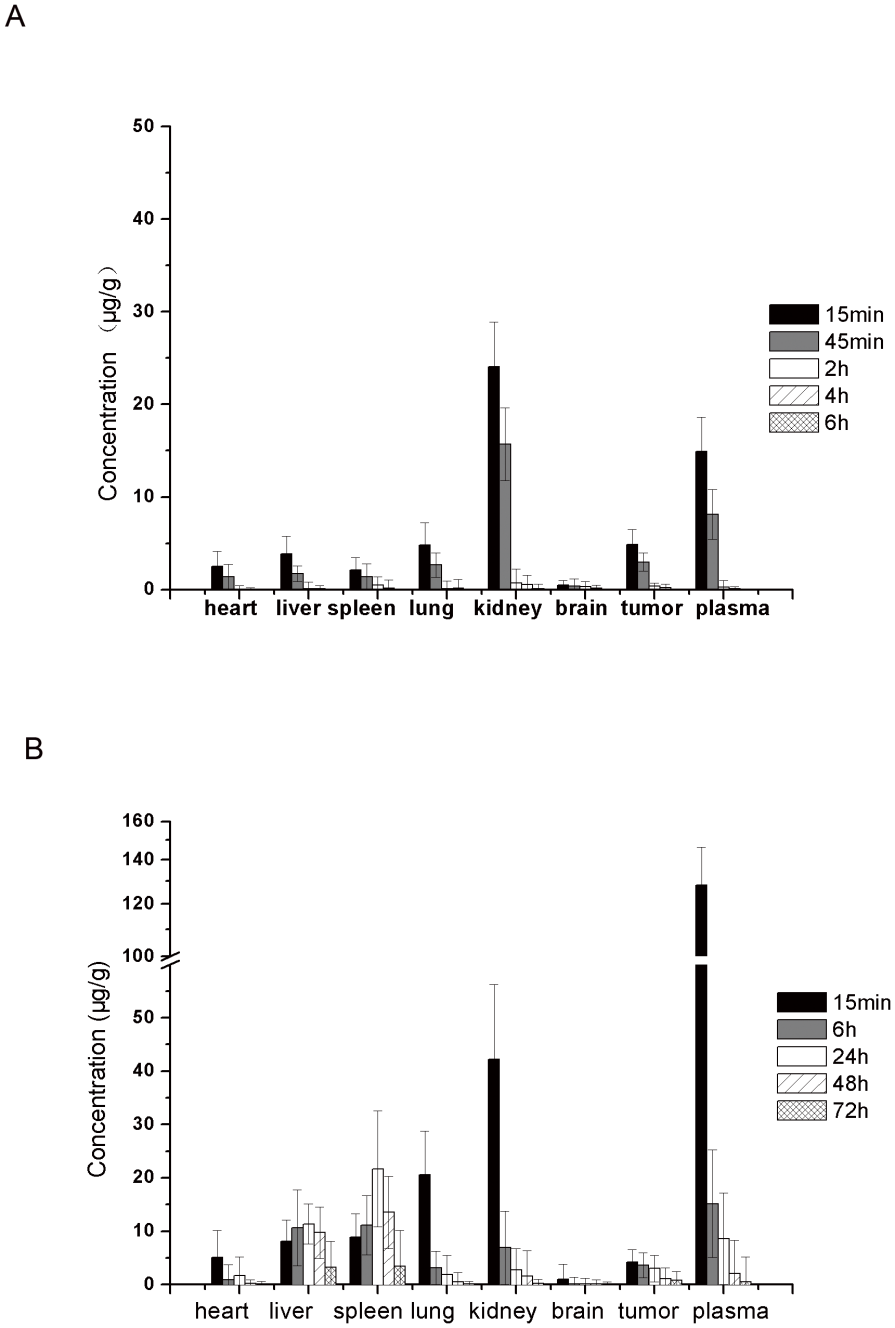
Biodistribution of 5-FA (A) and 5-FA-PAE (B) after *i.v.* injection. The tumor-bearing animal model was established by subcutaneous injection of H22 cells into Kunming mice. The control group and the test groups were administered intravenously with 20 mg/kg of 5-FA or 5-FA-PAE (equivalent to 20 mg/kg of 5-FA), respectively. The mice were exsanguinated and sacrificed at predetermined time points. Tissues (heart, liver, spleen, lung, kidney, brain and tumor) were collected, weighed and homogenized with two fold concentrated physiological saline. The samples of the test group were treated as hydrolyzed samples, whereas those of the control group were treated as unhydrolyzed samples. All data are presented as the concentration of 5-FA. Each value represents the mean ± standard deviation (n = 6).

Small molecular drugs eliminate quickly after intravenous administration, which could distribute them to normal tissues through capillaries nonspecifically. To analyze the *in vivo* biodistribution of 5-FA-PAE, the murine hepatic cancer cell line (H22) was used to establish the tumor-bearing animal model. The hepatoma H22 model has been widely use as a tumor model in the study of antitumor drugs and its mechanism. Generally, it is believed that the orthotopic tumor can better simulate the pathologic process of tumor development. However, due to the high mortality rate of animals and the complexity of operation, we adopted the ectopic model. After intravenous injection, at all time points, the concentration of 5-FA in kidney was significantly higher than other organs and in the tumor, suggesting that renal excretion was the major pathway of 5-FA elimination from the body. Other than kidney, 5-FA did not show any specificity in distribution with similar drug concentrations in heart, liver, spleen, lung and tumor. 15 min after the intravenous injection of 5-FA-PAE, the plasma concentration was the highest of all samples (128.1±18.3 µg/g). However, as the time increased, the plasma concentration of 5-FA-PAE decreased rapidly, dropping to 8,57±3.33 µg/g at 24 h, while the concentration in liver and spleen increased gradually and peaked at 24 h (11.35±3.78 µg/g in liver and 21.68±10.83 µg/g in spleen). 5-FA-PAE was detectable in all organs and tumor at 72 h after injection (consistent with the pharmacokinetics study), indicating that the retention time of 5-FA-PAE was longer than 5-FA (Fig. 4A and 4B).

**Figure 5:**
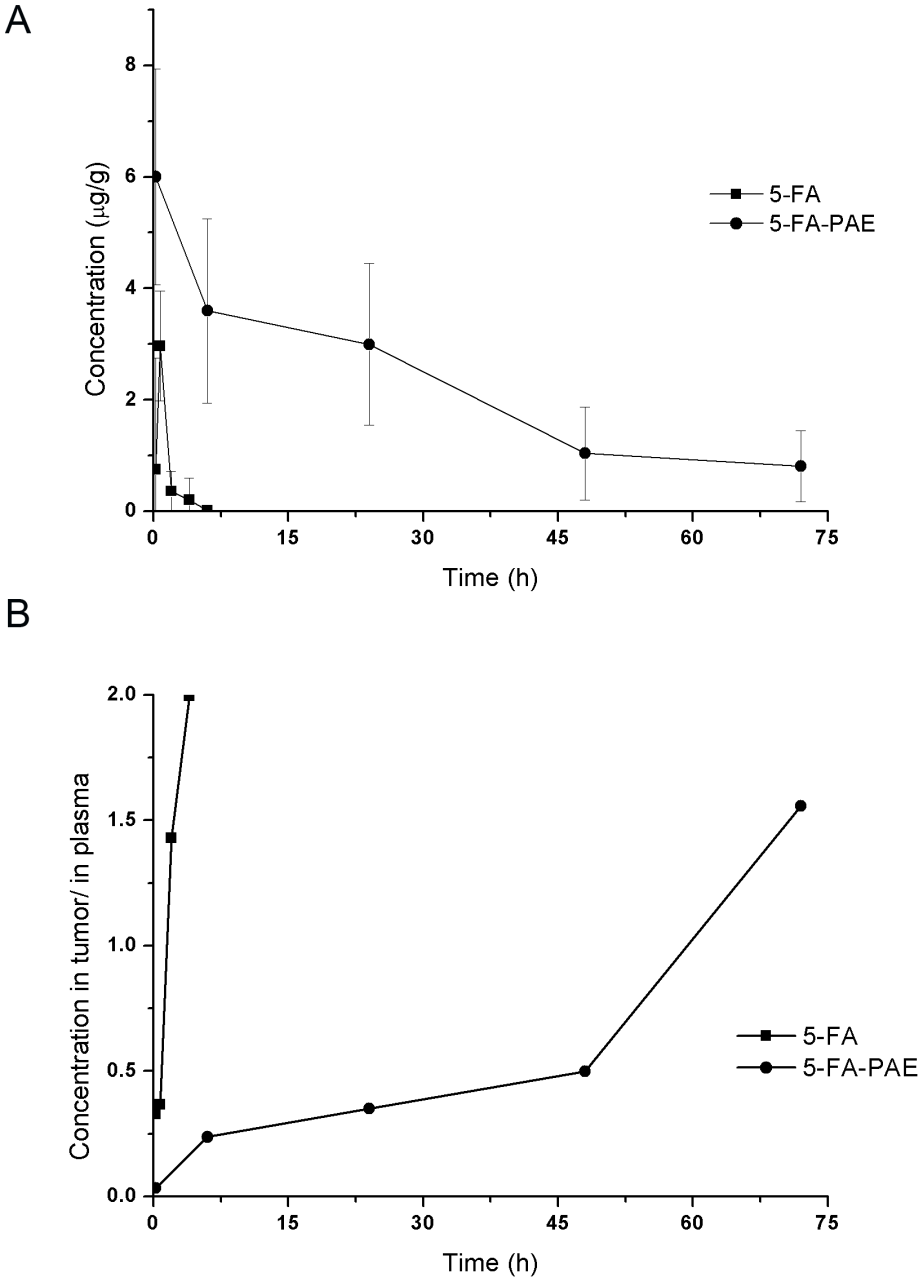
Drug concentration in tumor and plasma. The tumor-bearing mice model was described in the “*In vivo* biodistribution” section. The control group and the test groups were administered intravenously with 20 mg/kg of 5-FA and 5-FA-PAE (equivalent to 20 mg/kg of 5-FA), respectively. (A) Drug concentration of 5-FA and conjugated 5-FA-PAE in tumor at different time points. (B) Ratio of drug concentration in tumor vs. that in plasma of 5-FA and conjugated 5-FA-PAE.

The concentration of 5-FA and 5-FA-PAE in tumor is shown in Fig 5A. The concentration of 5-FA decreased rapidly after administration and was undetectable after 6 h. Though the maximum concentration of 5-FA-PAE was slightly lower than 5-FA at 15 min after injection (4.22±2.3 µg/g for 5-FA-PAE and 4.86±1.62 µg/g for 5-FA), the concentration of 5-FA-PAE remained at a relatively high level and lasted for 72 h, indicating that 5-FA-PAE could slowly release 5-FA in the tumor and exert an antitumor effect. The concentration in the tumor vs. those in plasma of 5-FA-PAE and 5-FA is displayed in Fig. 5B. 45 min after administration, the concentration of 5-FA in tumor was lower than that in plasma, and then increased rapidly afterwards. 4 h after injection, the ratio of tumor/plasma concentration reached 1.99, suggesting that 5-FA could distribute from plasma to tumor within a short time, and that the clearance rate of 5-FA in plasma was higher than that in the tumor. 6 h after administration, the 5-FA concentration was almost undetectable in both plasma and tumor. The ratio of tumor/plasma concentration of 5-FA-PAE increased steadily within 48 h after injection and was lower than 0.5. Between 48 h to 72 h, the ratio increased quickly and reached 1.56 at 72 h, indicating that the clearance of 5-FA-PAE from tumor was much lower than that from the plasma. These results indicated that the amount of 5-FA-PAE in tumor lasted longer compared with that of 5-FA, exhibiting a sustained-release profile.

Though the initial concentration and tumor/blood concentration ratio of 5-FA were higher than 5-FA-PAE, a high elimination rate of 5-FA severely limited its therapeutic effect in clinic. In comparison, the concentration of 5-FA-PAE in the tumor could be maintained at a relatively high level, which lasted for more than 70 h, despite the large variation (probably due to inter-individual difference). Similarly, the tumor/blood concentration ratio of 5-FA-PAE showed a gradually increasing trend.

### Antitumor effect in tumor-bearing mice

**Figure 6:**
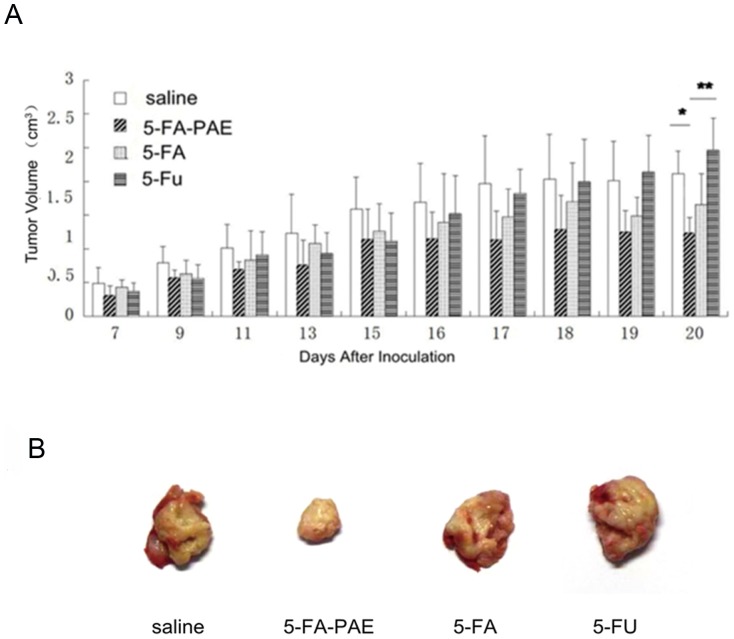
The antitumor effects on tumor-bearing mice. Mice were i.v injected with saline (20 mg/kg, 0.160 mmol/kg), 5-FA-PAE (284 mg/kg, 0.160 mmol/kg), 5-FA (30 mg/kg, 0.160 mmol/kg) or 5-Fu (20.47 mg/kg, 0.160 mmol/kg) on day 3, 5, 7, 9, 11, 13 and 15 after inoculation of H22 cells. On day 20, mice were sacrificed. Tumors and organs were removed and weighed. (A) The tumor volumes after inoculation (n = 6–12). * *p*<0.05, ** *p*<0.01. (B) Images of tumors in tumor-bearing mice on day 20 after inoculation of tumor cells (n = 6).

5-Fu is the first-choice antimetabolite in the treatment of colon cancer and colorectal cancer. 5-FA, a derivative of 5-Fu, has been reported to be effective and safe [56]–[59]. To address the antitumor activity of the prodrug, 5-FA and 5-Fu were both used as controls. In the pharmacokinetics studies of anticancer drugs, two dosing regimens are commonly used. One is the preventive administration strategy in which drugs are administered at the beginning of the tumor growth. The other one is the therapeutic administration with drugs administered when the tumor growth reached a certain size. Since the relatively high mortality rate of the H22 tumor model in the later period of this experiment, we adopted a prophylactic administration scheme, i.e. 72 h after inoculation, mice with no signs of tumor growth were excluded from this experiment. Based on the pharmacokinetics and biodistribution results, we administered the drugs every other day (from day 3 to day 15 after inoculation). The antitumor effect of 5-FA-PAE was assessed by analyzing tumor volume, tumor control rate and the organ/body weight index of tumor-bearing mice. From the beginning of administration, the tumor volume of 5-FA-PAE group was smaller than that of the saline group, showing the highest antitumor activity (Figure 6A and 6B). The 5-FA and 5-Fu groups also displayed some antitumor effect. However, after the last administration on day 15, the tumor volume of these two groups increased obviously, while the tumor size of the 5-FA-PAE group did not, which suggested that the antitumor activity of 5-FA-PAE could last for a longer time. This is compatible with the pharmacokinetics results in which 5-FA-PAE showed a much longer retention time than that of 5-FA. 20 days after inoculation, the average tumor volume of 5-FA-PAE group was significantly smaller than that of the 5-Fu and saline groups (*p*<0.01). However, no significant differences were observed between the 5-FA and 5-FA-PAE groups. This may be due to the large variation of the 5-FA group.

Though the tumor control rates of the 5-FA-PAE and 5-FA groups were not significantly different (*p*>0.05), the tumor control rates of the 5-FA-PAE group (51.9±11.2%) and the 5-FA group (35.0±17.6%) were significantly higher than that of the 5-Fu and saline groups (Table 4). Since the tumor growth can affect the weight of normal organs, the organ/body weight index was used to assess the impact. The tumor/body index of the 5-FA-PAE (3.381±1.224) group was much lower than those of the 5-Fu (8.816±3.578) and saline groups (7.088±1.961, *p*<0.05, Table 5). No significant differences were observed in other organ/body indices.

**Table 4:** Tumor weight and tumor control rate of mice administrated with saline, 5-Fu, 5-FA or 5-FA-PAE.

Treatment	Tumor weight (g)	Tumor control rate (%)
saline	2.229±0.521	-
5-FA-PAE	1.072±0.249 **	51.9±11.2Δ
5-FA	1.449±0.392 *	35.0±17.6Δ
5-Fu	2.383±0.841	−6.9±37.7

*p<0.05 vs. saline group.

**p<0.01 vs. saline group.

Δ *p*<0.05 vs. 5-Fu group.

Each value represents the mean ± SD (n = 6).

**Table 5:** The organ/body weight index of mice administrated with saline, 5-Fu, 5-FA or 5-FA-PAE.

Tissue	saline	5-FA-PAE	5-FA	5-Fu
Heart	0.440±0.047	0.431±0.042	0.443±0.031	0.424±0.038
Liver	5.643±0.291	6.255±1.023	5.737±1.068	5.601±0.897
Spleen	1.226±0.390	1.360±0.543	1.398±0.478	1.264±0.694
Lung	0.978±0.236	0.955±0.141	1.068±0.407	0.900±0.109
Kidney	1.368±0.131	1.377±0.083	1.426±0.113	1.298±0.089
Brain	1.355±0.110	1.317±0.203	1.421±0.302	1.452±0.308
Thymus	0.192±0.097	0.261±0.070	0.221±0.155	0.167±0.086
Tumor	7.088±1.961	3.381±1.224* Δ	4.957±2.336	8.816±3.578

*p<0.05 vs. saline group.

Δ *p*<0.05 vs. 5-Fu group.

Each value represents the mean ± SD (n = 6).

Owing to the conjugation with PEG, 5-FA-PAE exhibited a longer retention time, which led to a long-lasting antitumor effect. Notably, during the administration period, the death rate in the tumor-bearing mice of the 5-FA-PAE group is relatively high. This is probably due to the tumor growth and the toxicity of 5-FA-PAE, which is also a drawback of our present regimen and needs further refinement. However, after administration of all doses, no more deaths were observed in the 5-FA-PAE group, indicating that the toxicity caused by repeated administration of 5-FA-PAE was reversible. While in the 5-FA and 5-Fu groups, large number of animal deaths were observed after all administrations, suggesting a shorter duration of their antitumor effect.

## Conclusion

To solve the paradox of drug loading and the molecular weight of PEG, we synthesized a PEG multi-hydroxyl derivative (PAE). PAE was coupled with 5-FA via ester bonds to afford 5-FA-PAE, and the drug loading efficiency was shown to be 10.8-fold higher than using unmodified PEG. Besides, the retention time and bioavailability of 5-FA-PAE were greatly improved compared to 5-FA, showing a prolonged half-life and improved antitumor efficacy *in vivo*. Owing to the improved drug loading efficiency and prolonged half-life, the multi-hydroxyl PEG derivative PAE proves to be an efficient carrier for 5-Fu. Future study should focus on further improving the tumor-targeting efficiency and the antitumor effect of 5-FA-PAE while reducing its toxicity. This paper provides some insights for the future development of antitumor drugs using PEG as a drug carrier.

## Supporting Information

File S1 **Supporting files. Figure S1**, Identification of different polymers. The ^1^H-NMR spectra of 5-Fu (A), 5-FA (B), PEG (C), the polymeric carrier PAE (D) and the prodrug 5-FA-PAE (E). **Table S1**, The number of blood platelets in mice administered with saline, 5-Fu, 5-FA, PAE or 5-FA-PAE. (×10^9^/L). **Table S2**, Plasma concentration of 5-FA and 5-FA-PAE at different time points.
(DOC)
