# Supplementary material for: A Polymeric Prodrug of 5-Fluorouracil-1-Acetic Acid Using a Multi-Hydroxyl Polyethylene Glycol Derivative as the Drug Carrier
Source: PLoS One. 2014 Nov 12;9(11):e112888. doi: 10.1371/journal.pone.0112888 (PMC4229301; doi:10.1371/journal.pone.0112888)
Supplement: File S1 — Supporting files. Figure S1, Identification of different polymers. The 1H-NMR spectra of 5-Fu (A), 5-FA (B), PEG (C), the polymeric carrier PAE (D) and the prodrug 5-FA-PAE (E). Table S1, The number of blood platelets in mice administered with saline, 5-Fu, 5-FA, PAE or 5-FA-PAE. (×109/L). Table S2, Plasma concentration of 5-FA and 5-FA-PAE at different time points. (DOC) [file pone.0112888.s001.doc]

**Supporting Information**

**Figure S1**

**A**


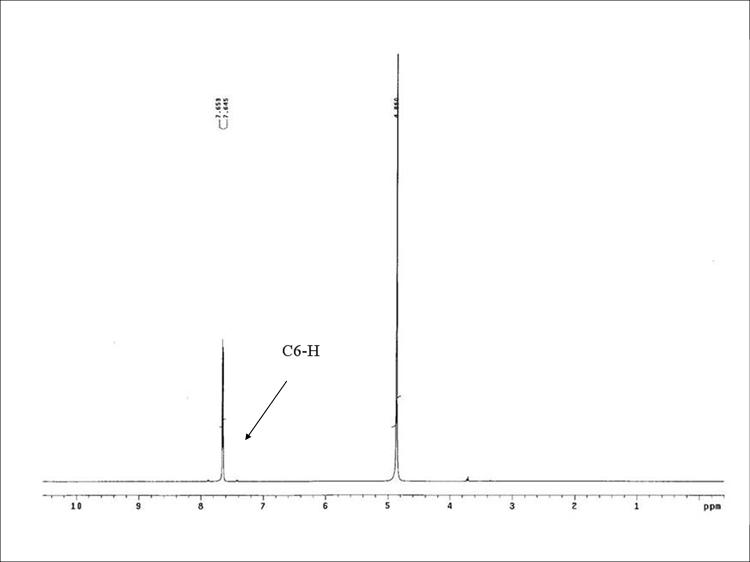


**B**


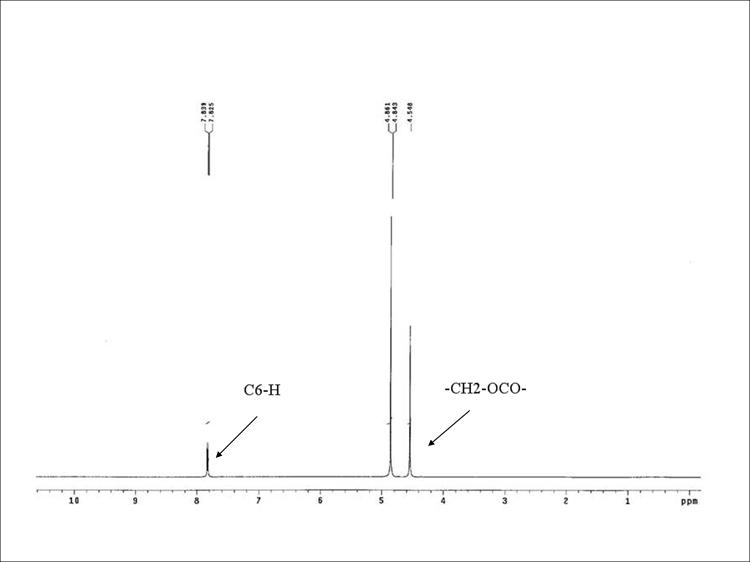


**C**


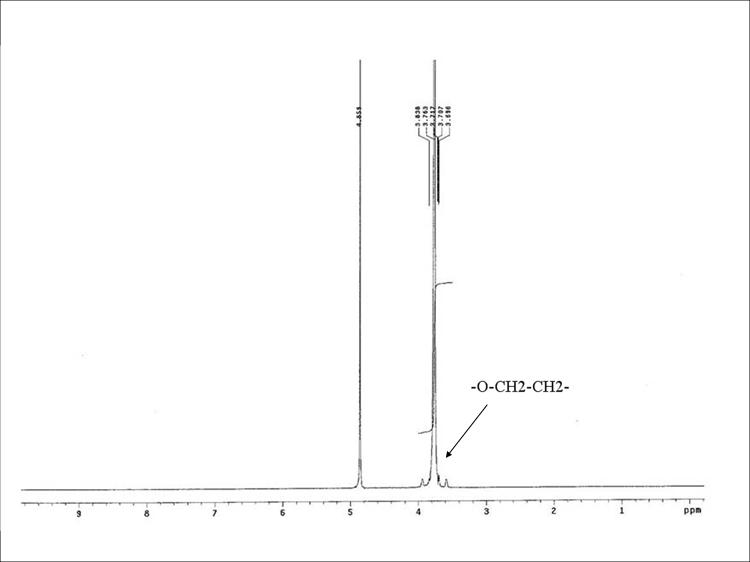


**D**


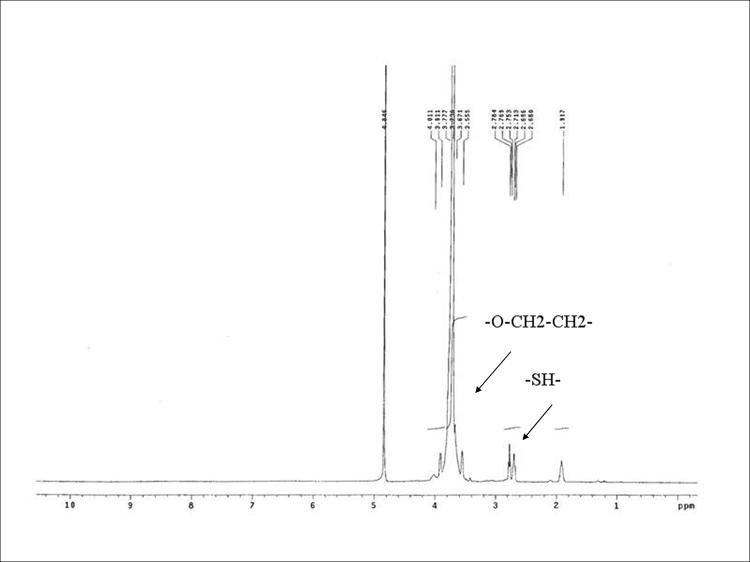


**E**


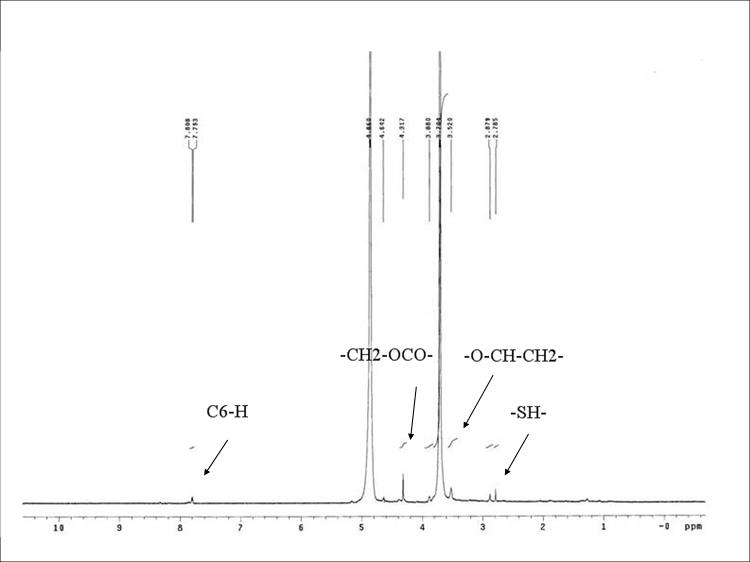


**Table S1.** The number of blood platelets in mice administered with saline, 5-Fu, 5-FA, PAE or 5-FA-PAE. (×10^9^/L)

| time  group | 1 day before injection | 1 day after injection | 4 days after injection | 7 days after injection | 10 days after injection |
| --- | --- | --- | --- | --- | --- |
| saline | 874 ± 189 | 871 ±113 | 804 ± 139 | 887 ± 155 | 805 ± 200 |
| 5-FA-PAE | 827 ± 169 | 826 ± 199 | 811 ± 146 | 815 ± 184 | 823 ± 146 |
| 5-FA  PAE | 829 ± 190  839 ± 174 | 945 ± 139  804 ±189 | 781 ± 250  736 ± 208 | 822 ± 198  849 ± 176 | 860 ± 188  815 ± 167 |
| 5-Fu | 876 ± 189 | 792 ± 122 | 819 ± 211 | 858 ± 254 | 863 ± 382 |

Each value represents the mean ± SD (n=12).

**Table S2.** Plasma concentration of 5-FA and 5-FA-PAE at different time points.

| **Time 5-FA (μg/ml)** | | **5-FA-PAE (μg/ml)** | |
| --- | --- | --- | --- |
|  |  | **Total Concentration Free 5-FA** | |
| 1 min  5 min  10 min  15 min  30 min  45 min  1 h  2 h  4 h  6 h  8 h  10 h  24 h  48 h  72 h  96 h | 79.917 ± 10.158  36.861 ±2.858  28.311 ± 3.516  19.502 ±5.071  8.934 ± 2.801  4.508 ± 1.677  2.645 ± 0.986  1.149 ± 0.814  0.271 ± 0.039  0.162 ± 0.056  0.170 ± 0.077  -  -  /  /  / | /  236.179 ± 11.042  /  183.369 ± 10.408  136.876 ± 6.469  /  91.372 ± 7.271  55.218 ± 8.710  26.901 ± 3.808  17.699 ± 1.966  /  9.388 ± 0.667  2.544 ± 0.229  0.633 ± 0.069  0.323 ± 0.036  0.220 ± 0.152 | /  7.903 ± 3.327  /  6.496 ± 3.906  5.189 ± 3.036  /  3.461 ± 0.928  2.646 ± 0.651  1.222 ± 0.324  0.692 ± 0.223  /  0.407 ± 0.221  0.105 ± 0.029  0.113 ± 0.039  0.118 ± 0.016  0.085 ± 0.110 |

- undetectable

**/** no plasma sample taken at this time point

|  |
| --- |
